# Calcium Signalling in Breast Cancer Associated Bone Pain

**DOI:** 10.3390/ijms23031902

**Published:** 2022-02-08

**Authors:** Andrea Bortolin, Estrela Neto, Meriem Lamghari

**Affiliations:** 1 i3S—Instituto de Investigação e Inovação em Saúde, Universidade do Porto, Rua Alfredo Allen 280, 4200-135 Porto, Portugal; andrea.bortolin@i3s.up.pt (A.B.); estrela.neto@ineb.up.pt (E.N.); 2 INEB—Instituto de Engenharia Biomédica, Universidade do Porto, Rua Alfredo Allen 280, 4200-135 Porto, Portugal; 3 FEUP—Faculdade de Engenharia da Universidade do Porto, Rua Dr. Roberto Frias s/n, 4200-465 Porto, Portugal

**Keywords:** calcium, signalling, nociception, pain, cancer, bone, metastasis, allodynia, hyperalgesia

## Abstract

Calcium (Ca^2+^) is involved as a signalling mediator in a broad variety of physiological processes. Some of the fastest responses in human body like neuronal action potential firing, to the slowest gene transcriptional regulation processes are controlled by pathways involving calcium signalling. Under pathological conditions these mechanisms are also involved in tumoral cells reprogramming, resulting in the altered expression of genes associated with cell proliferation, metastatisation and homing to the secondary metastatic site. On the other hand, calcium exerts a central function in nociception, from cues sensing in distal neurons, to signal modulation and interpretation in the central nervous system leading, in pathological conditions, to hyperalgesia, allodynia and pain chronicization. It is well known the relationship between cancer and pain when tumoral metastatic cells settle in the bones, especially in late breast cancer stage, where they alter the bone micro-environment leading to bone lesions and resulting in pain refractory to the conventional analgesic therapies. The purpose of this review is to address the Ca^2+^ signalling mechanisms involved in cancer cell metastatisation as well as the function of the same signalling tools in pain regulation and transmission. Finally, the possible interactions between these two cells types cohabiting the same Ca^2+^ rich environment will be further explored attempting to highlight new possible therapeutical targets.

## 1. Introduction

A broad variety of cellular functions are tightly regulated by Ca^2+^ intracellular signals. Some of them are part of the fastest signalling systems in the human body, like activation of exocytosis at synaptic junctions and muscle contraction, operating at a microseconds time scale. Other mechanisms taking place within minutes to hours, like gene transcription regulation and cell proliferation are slower but still of fundamental importance for cellular life, especially in the context of cancer evolution. For this reason, calcium signalling lays also behind many breast cancer tumoral mechanics [1].

Breast cancer represents a major concern worldwide, being the second leading cause of death for tumour, despite the high remission rate in case the disease is timely identified and treated before breast cancer advancement and bone metastasis [2,3]. Breast cells are intrinsically linked to calcium, being involved in physiological calcium homeostasis during lactation. As a consequence of their sensitivity to calcium concentration, calcium signalling mechanisms mainly modulating calcium channels and pumps expression, rule breast cancer dynamics like cells proliferation, metastasis, death and drug resistance [1,2]. In the context of breast cancer disease progression, cell proliferation and death control are extremely important, as well as their capacity to home to a different body site.

In approximately 75% of advanced breast cancer cases, breast cancer tumoral cells spread to the bone. Their presence in this environment leads to the alteration of the delicate bone signalling niche, resulting in a pathological cancer-associated bone pain (CABP) in more than 70% of patients. In the bone metastatic niche, cohabited by breast cancer and bone resident cells, tumoral cells completely alter the delicate paracrine signalling responsible for bone homeostasis, with a net imbalance toward bone resorption [3]. Together with this, neuronal cells seem to have a relevant role in tumoral cells homing to the bone while breast cancer cells can directly and indirectly foster neuronal axons ingrowth in a feed-forward loop [4]. The newly-generated signalling mechanics leading to bone resorption and micro-fractures, combined with the augmented presence of nociceptive neuronal fibres, result in increased painful cues from the nociceptive neurons and therefore in breast cancer associated bone pain. CABP is a combination of background, spontaneous and incident pain. Background pain is described as dull and continuous, exacerbates as cancer progress and can be controlled with conventional analgesics. In contrast, spontaneous pain (occurring without any obvious precipitating event) and incident pain (evoked on movement of the tumour-bearing bone) are rapid in onset and of intermittent and transient nature. For this reason, they are also referred to as “breakthrough pain” due to the ability to overcome the analgesic therapeutic regimen, and their alleviation by mean of currently available drugs is unsatisfactory, and occasionally associated with dose limiting adverse effects. CABP is a major concern worldwide, reducing the individual’s functional status and quality of life, and bearing a high socio-economical cost. For these reasons, a deeper understanding of this pathology, comprising the signalling mechanisms connecting all the cellular players, is pivotal to achieve a conscious involvement in fighting it [3].

Calcium signalling mechanisms are also involved in pain transmission, where the Ca^2+^ ion has the upper hand in signal transduction along neuronal axons and in synaptic neurotransmitter release. Furthermore, besides the mere fast signal transduction, slower calcium signalling mechanisms are involved in cues processing, especially at the level of the central nervous system, with a consequent role in pain sensitisation and chronicization. In this complex context, breast cancer and bone pain seem to be connected with a narrow strand by calcium, pivotal player underlying a broad variety of signalling cascades going from the fast signal transmission in neurons to the slower migration and gene expression mechanisms in cancer cells [5].

This review will follow the intracellular calcium signalling systems as a common thread connecting breast cancer cells, neurons and the bone microenvironment in CABP context. We will first introduce the most explored calcium signalling pathways and the main calcium sources used by cells, together with the mechanisms maintaining calcium homeostasis at the cellular level. We will therefore go deeper in the so far known roles of calcium signalling mechanisms in breast cancer disease progression and in pain signalling and chronicization. Finally we will review the known mechanisms used from the cancer cells to modify the bone context, transforming it in a more favourable environment for tumour replication and survival. The role of tumoral cells in CABP will then be addressed, analysing the influence they have on the different cells involved in it, on bone micro-anatomy, on pain initiation and chronicization mechanisms.

## 2. An Overview on Calcium Signalling Mechanisms

Despite a huge diversity in terms of time scale and cell types, Ca^2+^ signalling molecular players are shared amongst all cells, but thanks to specific gene expression regulation, splicing variants and different spatial localisation patterns proper of every cell type, the activation of each precise mechanism can lead to completely different effects [6,7]. The level of intracellular calcium is determined at any time and space by the balance between the “on” cues, resulting in calcium release into the cytoplasm by mean of cell membrane and intracellular calcium stores, and the “off” effectors represented by membrane pumps, channels and cytoplasmic buffers removing the signal. In this context, small localised variations in the “on-off” reactions taking place near calcium-sensitive receptors, can be amplified and give rise to Ca^2+^ release from internal cellular stores and Ca^2+^ entry from the extracellular environment [6,7].

**Figure 1:**
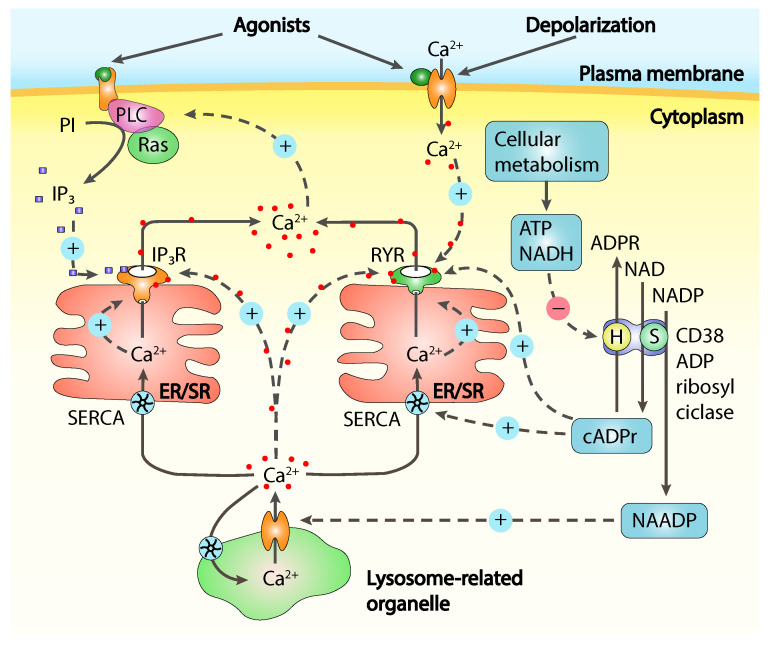
Schematic overview of the main Ca^2+^ signalling mechanisms. Calcium release from the intracellular calcium stores is mainly mediated by inositol-1,4,5-triphosphate receptors (IP_3_Rs) and ryanodine receptors (RYRs), sensitive to signals present both inside and outside the endoplasmic/sarcoplasmic reticulum (ER/SR). Inositol-1,4,5-triphosphate (IP_3_) is produced by different isoforms of phospholipase C (PLC) and binding to IP_3_Rs can trigger calcium release. The CD38 ADP ribosyl cyclase has both synthase (S) and hydrolase (H) functions producing cyclic ADP ribose (cADPr) and nicotinic acid adenine dinucleotide phosphate (NAADP) and has been proposed to act as a cellular metabolism sensor since ATP and NADH can inhibit the hydrolase enzymatic function. The exact way cADPr and NAADP influence calcium release is still unclear but they seem to indirectly act on both RYRs and IP_3_Rs. cADPr might enhance sarco(endo)plasmic reticulum Ca^2+^-ATPase (SERCA) pump activity, increasing the level of Ca^2+^ in the ER/SR, sensitising the RYRs. NAADP triggers calcium release from a channel located on lysosomes-related organelles, locally increasing the intracellular calcium concentration which can in turn result in direct stimulation of RYRs and IP_3_Rs or in an indirect stimulation through an increase of Ca^2+^ concentration in ER/SR similarly to what happens with cADPr. PI, phosphatidylinositol-4,5-bisphosphate. Adapted from [5].

Calcium release from intracellular Ca^2+^ stores, mainly represented by the endoplasmic reticulum (ER) or its muscular counterpart, the sarcoplasmic reticulum (SR), is often referred as store-operated Ca^2+^ release (SOCR) and is controlled by Ca^2+^ itself and from many molecular messengers [8] like inositol-1,4,5-triphosphate (IP${}_{3}$), cyclic ADP-ribose (cADPR), nicotinic acid adenine dinucleotide phosphate (NAADP) and sphingosine-1-phosphate (S1P), all concurring in calcium release through channels activation or modulation (Figure 1).

The extracellular space is the primary source of Ca^2+^ to replenish the intracellular stores and to maintain the intracellular basal calcium concentration. Especially in case of excitable cells, thanks to a large electrochemical gradient actively maintained across the cell membrane, Ca^2+^ waves cross the membrane activating many signalling cascades [9]. Ca^2+^ entry from the extracellular environment is under control of many different plasma membrane channels, in response to a broad variety of stimuli including depletion of intracellular stores, intracellular messengers, membrane stretch and depolarisation, noxious stimuli and extracellular signalling molecules. The most characterised channels permitting Ca^2+^ fast entry are the plasma membrane located voltage-operated calcium channels (VOCCs), able to control fast cellular processes like neurotransmitter exocytosis in neurons and muscle contraction. Another broadly studied class of membrane calcium channels is represented by receptor-operated channels (ROCs), triggered by extracellular signalling molecules like the N-methyl-D-aspartate receptors (NMDARs), while second-messenger-operated-channels (SMOCs) respond to internal stimuli like cyclic-nucleotides-gated channels found in sensory systems, and the arachidonic-acid-sensitive channels, activated by the intracellular increase of arachidonic acid, independently from cytoplasmic Ca^2+^ increase [10,11]. Other channels like the store-operated Ca^2+^ channels (SOCC), can be thermosensors or stretch-activated belonging to the large family of the transient receptor protein (TRP) non-selective calcium-permeable ion channels, the most important in noxious stimuli detection and transmission [5]. The TRP family comprises 28 members divided into six subfamilies, classified as canonical (TRPC), vanilloid (TRPV), ankyrin (TRPA), melastatin (TRPM), polycystin (TRPP), and mucolipin (TRPML) [12]. TRPs have a central role in mechanical, thermal (heat, cold, or both), chemical, and osmotic stimuli detection being both ligand gated and responding to intracellular calcium stores depletion depending on the specific subunit isoforms composing the channel. [13]. Thanks to their tendency to have a low conductance these channels normally operate at longer time scales if compared with VOCCs and are particularly important in slow cellular processes like cell proliferation, migration and, in case of pain, sensitisation [14].

## 3. Calcium Signalling in Breast Cancer

Cancer-related pathology is mainly a consequence of a deregulated, excessive cell proliferation both in the primary and secondary metastatic sites. The hallmarks of cancer disease vary depending on the disease stage, and fighting them means acting on different cellular mechanisms. Calcium signalling has turned out to be extremely important in tumoral cells thanks to its central role in signal transduction, transcription modulation and cell-to-cell communication. Amongst the plethora of channels and mechanisms involved in calcium signalling, several have been shown to play important roles in breast cancer cells proliferation, drug resistance, invasion and metastasis, gaining a particular interest as potential pharmacological targets [1].

### 3.1. Ca*^2+^* Channels

Several calcium channels have altered expression in breast cancer and some of them are specifically modulated in certain breast cancer molecular sub-types. Although Ca^2+^ signalling is important in proliferative mechanisms, while an effective calcium homeostasis is essential for survival, an overload of intracellular calcium can promote cancer cell death [15,16]. In the complex context of Ca^2+^ homeostasis within breast cancer cells, both activation or silencing of TRP membrane calcium channels can induce cell death, depending on the specific protein. TRPV6 is a highly Ca^2+^ selective and constitutively active ion channel [17], highly expressed in malignancies including breast cancer [18]. A correlation between TRPV6 DNA copy number and its RNA expression suggested a positive selection for this trait in some cancers evolution [18] and siRNA mediated TRPV6 silencing decreased breast cancer cells proliferation in-vitro and resistance to chemotherapeutics in the T47D oestrogen resistant human ductal carcinoma cell line [19]. Still, silencing of TRPV6 channels resulted in reduced migration and invasiveness of the human-derived triple-negative MDA-MB-231 cells in-vitro [20] and in increased apoptotic cell population in T47D breast cancer cells culture [19], similarly to what happens when exposed to TRPV6 inhibitor SOR-C13 [21]. Recent developments on TRPV6 specific novel inhibitors and understandings on different drug target sites in TRPV6 may aid in the design of new therapeutic strategies targeting TRPV6 in cancer [22,23,24,25].

In a similar way, TRPV4 are modestly permeable to Ca^2+^ but their overexpression corresponds to breast cancer-specific increased viability, correlated with poorer overall and distant metastasis-free survival [26], greatly reducing the number of metastasis when knocked down in a 4T1 triple-negative syngenic mouse model. This probably results from transcriptional and post-translational regulation mechanisms, affecting cell rigidity [26], AKT activation and E-cadherin remodelling [27], with direct consequences on cell adherence, extravasation and metastasis. TRPV4 was also found to be overexpressed in some basal breast cancers and in cell lines like the human-derived triple-negative metastatic adenocarcinoma MDA-MB-468 [28], where its potent activation via GSK1016790A selective agonist, was shown to induce cell death, such effect was reduced in cells with silenced expression of TRPV4 and absent in cells lines with decreased levels of expression or absence [28].

TRPM7 has also been recognised as independent marker of progression and metastasis if highly expressed [29] and its silencing and inhibition were shown to suppress breast cancer cell line proliferation in-vitro, while TRPM7 knockdown reduced metastasis level in a MDA-MB-468 xenograft mouse model [30].

TRPA1 has been recently shown to be overexpressed and associated with some breast cancer cells survival whereas silencing it in the HCC1569 human epitelial metaplastic carcinoma cell line, reduced their ability to survive H_2_O_2_ treatment through an up-regulation of anti-apoptotic and pro-survival pathways mediated by a Ca^2+^ influx mechanisms promoting reactive oxygen species tolerance [31].

An important role in breast cancer survival against chemotherapeutics has been identified for TRPC5, as the regulator of the multi-drug resistance (MDR)ATPase 1 [32,33]. The human oestrogen receptor positive (ER^+^) MCF-7 breast cancer cell line feature high levels of TRPC5 expression making them resistant to doxorubicin cytotoxic effect [32], and TRPC5 silencing can increase sensitivity to doxorubicin by reducing (MDR)ATPase 1 expression. Interestingly, chemotherapy treated patients, have elevate levels of TRPC5 in the circulating extracellular vesicles where a higher level is associated with a poor response to the therapy [33]. In this context it has been hypothesised that the circulating extracellular vesicles coming from the highly expressing TRPC5 cells are transferred to the cells expressing lower levels of (MDR)ATPase 1 increasing its transcription by activating the Ca^2+^-dependent transcription factor NFAT, therefore promoting drug resistance in a positive feedback loop [33].

Another type of channels involved in calcium signalling in breast cancer are the calcium release-activated channels comprising Orai1 subunits, implicated in Ca^2+^ entry into mouse mammary epithelial cells through the basolateral membrane and seem to have a role in myoepithelial cells contraction for milk expulsion in physiological conditions [34]. Orai1 and 3 play different roles in distinct breast cancer subtypes since Orai1 signalling pathway alterations may be characteristic of poorest prognosis in basal cancer subtype [35]. In ER^+^ cancer cell lines like MCF-7 it has a role in SOCE and silencing it results in reduced cells proliferation in-vitro and in-vivo [35,36] as well as in a reversion of the antiapoptotic effects of collagen in MCF-7 and T47D cells [37]. Orai3 is selectively required in MCF-7 cells, it is a contributor in SOCE in ER^+^ but not in ER_-_ breast cancer cells and silencing Orai3 results in reduced proliferation, decrease in anchorage-independent growth and decreased xenograft development in-vivo [38,39,40,41]. However, Orai1 knockdown in MCF-7 cells has also been shown to drastically reduce SOCE in MCF-7 cells [42]. Again, Orai3 overexpression, protects T47D cells against cell death inducers like cisplatin, fluorouracil, paclitaxel, staurosporin and thapsigargin, suggesting a role for Orai3 in breast cancer resistance to chemotherapeutics [43]. Interestingly Orai1 seems to exert its role also on the extracellular tumoral environment. The human micro-vascular endothelial cell line of tumour angiogenesis (HMEC-1), if cultured in presence of MDA-MB-231 and BT-549 (ER^−^, human epidermal growth-factor receptor 2 negative (HER2^−^) breast ductal carcinoma) conditioned medium shows an enhanced angiogenic induction which is in turn significantly reduced upon Orai1 silencing, highlighting a role for Orai1 in Ca^2+^-mediated release of pro-angiogenic factors in triple negative breast cancer cell lines. Advanced analogues of 2-Aminoethoxydiphenyl borate (2-APB), a concentration-dependent SOCE modulator, were shown to block SOCE in MDA-MB-231 cells reducing their viability [44].

Inositol-1,4,5-triphosphate receptors (IP${}_{3}$Rs) exert an important function in calcium signalling in breast cancer thanks to their role in cell proliferation regulation, since G protein-coupled receptors (GPCRS) and tyrosine kinase receptors coupled to phospholipase C (PLC) are main players in pro-proliferative signals initiation. By the way the IP${}_{3}$Rs contribution could go beyond the mere receptor activation. IP_3_R3s have been shown to be positively regulated on MCF-7 cell line by 17β-oestradiol, acting on oestrogen receptor as pro-proliferating signal, but IP_3_R3s silencing resulted in reduced proliferation via cell cycle arrest in the G0/G1 phase [45,46]. Double silencing of IP_3_R1 and IP_3_R3s isoforms resulted to be cytotoxic in several breast cancer cell lines but not in non-tumorigenic cells, while inducing the autophagy marker LC3-II in all of them pointing out an increased propensity for tumorigenic cell lines to die in response to an autophagy related pathway activation [47].

### 3.2. Calcium Pumps

Calcium channels can only take advantage of Ca^2+^ gradients across membranes but active transport proteins can make the difference where calcium physiological concentration can be altered both outside and inside cells. During lactation calcium is actively secreted into the milk and in physiological conditions plasma-membrane Ca^2+^-ATPase (PMCA) 2 is located on the apical membrane and is responsible for this direct Ca^2+^ efflux.

Under malignant conditions PMCA calcium pumps have been associated to breast cancer outcomes since PMCA2 has been positively associated with HER2 expression in human ductal carcinoma in situ and PMCA2 null mice develop less tumours when crossed with a HER2 cancer mouse model, indicating that it may be particularly important in HER2^+^ breast cancers growth [48]. Its role is confirmed in-vitro, since silencing of PMCA2 in MDA-MB-231 breast cancer cells reduces their proliferation, coadiuvate the antiproliferative effect of doxorubicin [49] and increases the necrosis induced by ceramide or ionomycin [46,50]. In contrast, PMCA2 overexpression reduced the sensitivity to ionomycin mediated apoptosis in T47D cells [51] and induced cell death in in-vitro cell lines [52].

The secretory pathway Ca^2+^ ATPase 2 (SPCA2) is a calcium pump located on Golgi significantly increased during lactation and involved in the secretion of Ca^2+^ into milk [53]. Its been proven to promote cellular proliferation also in a non-tumorigenic breast cell line probably through Orai1-mediated calcium influx activation [36] and SPCA2 silencing reduces MCF-7 cells proliferation in-vitro and in-vivo. SPCA channels may also exert their function on some Ca^2+^ sensitive enzymes in the Golgi impairing the proper folding of some growth factor receptors as also SPCA1 silencing reduced the proliferation in MDA-MB-231 cell line [54].

Sarco(endo)plasmic reticulum Ca^2+^-ATPase (SERCA) pumps are extremely important for calcium homeostasis in breast cancer cells. SERCA inhibition leads to the reduction of endoplasmic reticulum Ca^2+^ level which in turn results in a sustained unfolded protein response [55] while in MDA-MB-231 cells it induces cell death by excessive autophagy activation [56].

### 3.3. Mitochondria in Ca*^2+^* Signalling

Mitochondria play a central and multifunctional role in malignant tumour progression and death. Being involved in energy supply, red-ox homeostasis, oncogenic signalling and apoptosis, they are also an active component of the Ca^2+^ signalling network [57]. The mitochondrial uniporter (MCU) is a critical regulator of mitochondrial Ca^2+^ levels and is highly expressed in ER-negative tumours compared with the ER^+^, particularly in those with a basal-like phenotype [58]. It is part of a pre-defined gene set under the regulation of hypoxia inducible factor 1 alpha (HIF-1α) in breast cancer and there is a positive correlation between the expression of HIF-1α and MCU in-vitro seemingly found also in clinical data [59]. Advanced disease clinical samples were associated to high MCU levels and MCU silencing in MDA-MB-231 triple-negative human breast cancer cells, greatly reduces the size of the primary tumour in xenografts. Interestingly, this effect is not relevant on breast cancer cells in-vitro proliferation, suggesting that it may depend on the context and by environmental factors [59]. On the opposite hand, silencing MCU reduces the migration and the invasion capacities in MDA-MB-231 cells [59,60], impairing their ability to metastasise to other sites in-vivo [59].

### 3.4. Calcium-Sensing Receptor

During lactation a G protein-coupled receptor, calcium-sensing receptor (CaSR), in response to extracellular physiological calcium concentrations and to other organic and inorganic cations, plays a relevant role in calcium homeostasis [61]. CaSR is expressed in normal breast epithelial cells and when active, it inhibits parathyroid hormone-related protein (PTHrP) secretion, participating in calcium metabolism. PTHrP promotes the release of calcium from the bone tissue so, in presence of high Ca^2+^ circulating level, a reduction in PTHrP blood secretion corresponds to the inhibition of calcium release into blood circulation [62]. CaSR is also involved in regulation of calcium pumps and channels such as PMCA2, SPCA2 and ORAI1 [62]

Besides being expressed in normal breast epithelial cells, CaSR is expressed also in breast cancer cells. The exact role of CaSR in tumour promotion or suppression is still debated. Some studies report no effect on tumoral cells proliferation [63] while in other studies increased proliferation, migration and tumour progression promotion were shown [62]. In contrast, Chakrabarty laboratory reported proliferation and invasion inhibition together with enhanced sensitivity to cell death upon CaSR activation [64]. Despite the role of CaSR is still not clear, it affects diverse breast cancer cells traits such as proliferation, migration and death, and contributes to the pathophysiology of osteolytic bone metastasis [65] making it an interesting subject for further studies.

## 4. Calcium Signalling in Pain

Acute physiological pain, as an unpleasant sensory response to noxious stimuli, is essential to avoid potential injuries and for animals survival. Differently, pathological pain persists following the original stimulus or injury and can lead to suffering with a decrease in patients life quality, along with a high socio-economic cost.

The pain signalling pathway originates in the tissues densely innervated by peripheral primary sensory neurons like skin, viscera and bones, with their cell bodies situated within the dorsal root ganglia (DRG) in the intervertebral foramina. The noxious signal travels upward to the central nervous system (CNS) through unmyelinated C fibres and myelinated Aδ fibres to the spinal cord dorsal horn laminae I II and V where second order neurons project to the somatosensory and limbic system of the cortex via the spinothalamic and spinoreticulothalamic relays, where the nociceptive and the emotional/aversive components of pain are respectively processed. Together with this, descending pain modulating pathways (signals coming from the brainstem, hypothalamus and cortical structures) can modulate sensory inputs from primary afferent fibres and from neurons in the dorsal horn [14].

**Figure 2:**
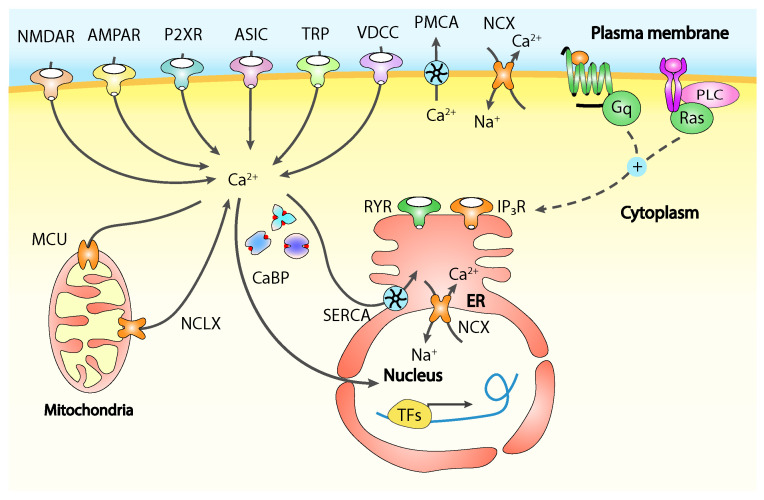
Calcium sources and sinks. Calcium can enter neurons from the extracellular space through many membrane channels as NMDARs and AMPARs, P2XRs, ASICs, TRP channels and VDCCs while PMCA pumps and NCX Ca^2+^ exchanger extrude calcium to the extracellular environment to maintain the basal membrane potential and regulating the intracellular calcium concentration. The activation of menbrane Gq-coupled GPCRs and TKRs can trigger calcium release from the ER via IP_3_Rs and ryanodine receptors (RYRs) where again SERCA and NCX are responsible for calcium re-uptake. Mitochondrial NCXL and MCU contribute to cytoplasmic calcium signalling by releasing and removing it respectively, while all calcium binding proteins (CaBP) act as calcium buffers amongst all stimuli, dynamically releasing and removing calcium ions depending on the concentration. Through nuclear pores cytosolic calcium can enter the nuclear space where it can regulate the activity of transcription factors acting therefore on genes expression. Adapted from [14].

Calcium is involved in functional and structural neuronal plasticity underlying peripheral and central sensitisation at all levels, while changes in the Ca^2+^ intracellular concentration are mediated by different channels including ligand-gated receptors, acid-sensing ion channels triggered by extracellular protons, ATP-responsive receptors, TRP channels and VOCCS, acting some times in different ways depending on the neuronal cell type and on their location on it, whether it is the cell body or one of the two axonal terminals of a primary sensory neuron (Figure 2) [14].

Abnormal neuronal Ca^2+^ homeostasis and calcium signalling are associated to numerous central nervous system disorders, including pathological pain diseases [14]. For example, in neuropathic pain, the mechanisms involving increased excitability and enhanced spontaneous activity of primary afferent neurons are strongly associated with a decrease in several aspects including voltage dependent calcium currents, recruitment of Ca^2+^ sensitive potassium channels, resting calcium concentration and in the magnitude of the evoked calcium transients in DRG neurons [66,67,68].

### 4.1. NMDARs and AMPARs

Intracellular calcium concentration both in peripheral and central neurons can be controlled by calcium entry through ligand-gated channels such as N-methyl-D-aspartate receptors (NMDARs) and calcium-permeable α-amino-3-hydroxyl-5-methyl-4-isoxazolepropionate receptors (AMPARs), responding to synaptically released neurotransmitters and membrane potential. NMDARs and AMPARs are considered key triggers for activity-dependent changes in neuronal excitability and function, while their altered expression and/or function has been observed in primary sensory neurons, spinal cord dorsal horn and in supraspinal regions under peripheral and central sensitisation [14].

Thanks to their calcium permeability, NMDARs constitute a significant source of synaptic activity-dependent calcium rises, playing a role in the regulation of several enzymes involved in signal transduction like kinases and phosphatases. For this reason, NMDARs are implicated in almost all aspects of pain plasticity [69,70,71], resulting in an attractive target for pain treatment.

AMPARs can also participate in postsynaptic calcium rises and calcium-dependent plasticity according to their subunit composition. More precisely, AMPARs missing the GluA2 subunit are characterised by an increased Ca^2+^ permeability and total calcium conductance and are particularly associated with signalling mechanisms underlying pain chronicization [14]. AMPARs located on peripheral pain-sensing neurons are at least partially implicated in pain chronicization since deletion and pharmacological inhibition of calcium-permeable AMPARs resulted in decreased hypersensitivity in models of chronic inflammatory pain and arthritis in-vivo with a loss in nociceptive plasticity in-vitro [72,73,74]. Finally, spinal cord located AMPARs are important for activity-dependent changes in synaptic processing of nociceptive stimuli [75] since neurons of the dorsal horn express a high level of calcium-permeable AMPARs [14] and an increased expression at postsynaptic sites enhances postsynaptic excitation and calcium influx, strengthening the AMPAR-mediated component of spinal synaptic transmission [14].

### 4.2. P2XRs

Ligand-gated, ATP-sensitive P2X purinoreceptors (P2XRs) are non-selective cation channels, which activation can lead to the downstream stimulation of VOCCS and Ca^2+^-dependent MAP kinases [76,77]. P2XRs subtypes P2X_1_, P2X_2_,P2X_3_ and P2X_4_ are particularly expressed in the peripheral nociceptive neurons but also in the dorsal horn, resulting therefore in increased excitability upon ATP stimulation on these cells [78,79]. P2XRs are sensitive to ATP concentrations changes to the nanomolar range and ATP activation of pain pathways has been well-documented as resulting in neuronal depolarisation and increased excitability [80,81]. ATP exerts a double function in this context since it has a stimulatory effect activating nociceptive cues in the peripheral neurons, but it is also released by the peripheral neurons at the dorsal horn junction resulting in an increased net nociceptive output [81].

There is a well-established correlation between peripheral and central sensitisation in inflammatory and neuropathic pain models and an increased activity and expression of P2XRs. P2X3 receptors-mediated currents and calcium transients were shown to be decreased upon anti-nerve growth factor (NGF) treatment, while NGF and calcitonin gene-related peptide (CGRP) treatment enhanced P2X3 receptor activity through diverse intracellular signalling pathways, by enhancing P2X3 expression and trafficking to the neuronal membrane. Together with the upregulation comes an increased ability of the receptor for being repeatedly activated by extracellular ATP, resulting in long-lasting painful signalling. Interestingly, since thy rely on different intracellular signalling mechanisms, singularly blocking NGF or CGRP signalling does not abolish P2X3Rs sensitisation, therefore suggesting a combined approach for pain treatment therapy [82,83].

Other signalling pathways could finally be impaired by P2XRs-induced calcium signals, contributing to mechanisms involved in pain sensitisation. This is the case of gamma-aminobutyric acid (GABA) receptor-dependent inhibition, where P2XRs seem to complex with GABA A receptors using them for co-trafficking to the cell surface, but GABA receptor degradation increased in presence of P2X2Rs agonist [84,85].

### 4.3. VOCCs

Voltage operated calcium channels are clearly involved in pain transmission [86]. Low-voltage activated (LVA) VOCCs (T-Type) are activated by action potentials close to the resting membrane potential making them an easily excitable target especially at level of neuronal dendrites [87,88] whereas on peripheral neurons they seem to be involved in pro-nociceptive functions. However, their involvement in chronic pain seems to alter depending on their expression site. In a neuropathic pain model, following chronic constriction injury, T-type VOCCs were upregulated in the spinal cord, where pharmacological silencing resulted in hyperalgesia and allodynia relief, but their activity in peripheral sensory neurons was drastically reduced, supporting the idea that, in neuropathic pain, T-type VOCCs upregulation may be important in central sensitisation while peripheral sensitisation may be driven by their downregulation [68,89,90].

High-voltage-activated (HVA) (L-, N- and P/Q-Type) VOCCs face two main changes in sensory neurons, under different peripheral chronic pain situations. In case of peripheral inflammation and spinal nerve ligation a decrease in HVA modulated calcium currents was observed, leading to a increased neuronal excitability [14], with widening action potentials, maximal firing rate increase, and burst firing higher incidence, effects also observed under VOCCs antagonist administration [14]. Neurotransmitter release and synaptic strength are both regulated by action potentials duration and burst firing [14]. A decrease in VOCCs calcium currents could therefore lead to neurotransmitter release augmentation in the presynaptic region of the dorsal horn with an increased excitation of its secondary neurons [68], where a restoration of the normal calcium influx has been observed to rectify the hyperexcitability of nociceptive neurons.

Secondly, painful nerve injury is correlated to an increased α2δ1 HVA VOCCs auxiliary subunit expression [91], meanwhile the N-Type VOCCs were found in higher quantity at the synaptic junctions of the spinal cord dorsal horn in acute inflammation and spinal nerve ligation pain models [14]. α2δ auxiliary subunit, rather than having a direct effect on pore formation and gating, seems to act as a chaperone protein with a role in HVA calcium channels trafficking to synaptic locations and in VOCCs-exocytosis coupling with a consequent increase in presynaptic neurotransmitter release rate and postsynaptic excitation [14]. This mechanism of action is confirmed by several studies where, while an overexpression of α2δ1 leads to enhanced VOCCs activity in sensory neurons and hyperexcitability in their targets within the dorsal horn [92], α2δ1 knockout in-vivo resulted in a significant delay in mechanical hypersensitivity onset [93] and knockdown with antisense RNA reversed allodynia in several models of chronic pain [94,95,96] inhibiting cell surface trafficking of N-type calcium channels in-vivo [97]. Consistently with these observations, the anti-nociceptive action of gabapentin seems to derive from its ability to bind to α2δ1 disrupting VOCCs trafficking to the presynaptic sites in-vitro [98,99].

### 4.4. Endoplasmic Reticulum Calcium Release

One of the main sources of Ca^2+^ in neurons, besides the extracellular space, are the intracellular calcium stores mainly consisting in the ER [14], representing therefore an important signalling mediator whose characteristics are often altered under pathological pain conditions. The main activators of ER Ca^2+^ release associated with pain are ligand-operated receptors like tropomyosin receptor kinase A (TrkA) and TrkB responding respectively to NGF and brain-derived neurotrophic factor (BDNF) [100], P2Y purinoreceptors, group I/II mGluRs, serotonergic (5-HT) receptors, noradrenergic receptors, opioid receptors, and many others. These receptors normally have a G-protein-couple domain (GPCR) or receptor tyrosin kinase (RTK) domain responsible for the signalling cascade activation [101,102,103]. In response to these calcium releasing pathways other ion channels are activated like calcium-permeable acid-sensing ion channels, TRP channels, NMDARs, and P2XRs, but also calcium-sensitive chloride and potassium channels [14], making GPCRs and RTKs interesting potential therapeutical targets for peripheral and central pain sensitisation [101,104].

An example is P2Y receptor activity which has been linked to neuropathic and inflammatory pain sensitisation [105,106] since they can sensitise TRPV1 channels and inhibit P2XRs and N-type VOCCs in peripheral sensory neurons [107,108,109].

SOCE is another mechanism of calcium influx regulated by the ER, although its role is still not completely clear. Upon nerve injury the function of SOCC was increased in DRG neurons and upon administration of a SOCC inhibitor, the thermal and mechanical sensitivity induced by spared nerve injury were reduced while hypersensitivity was delayed [110,111].

Ryanodine receptors (RYRs) main agonist is Ca^2+^ itself and they play an important role in the amplification of presynaptic Ca^2+^ signals leading to neurotransmitters release [112,113] and have been proposed to participate in pain chronicity both in nociceptive neurons via stimulation of calcium-/CaM-dependent protein kinase CaMKII [114] and in the spinal cord dorsal horn triggering the induction of long-term potentiation at C-fibre synapses [115,116,117].

### 4.5. PMCA

Plasma-membrane Ca^2+^-ATPase (PMCA)s are the main high-affinity Ca^2+^ pumps in neuronal cells responsible for its extrusion to the extracellular space and are therefore important for a fast restore of calcium concentration after intracellular Ca^2+^ rise, together with other mechanisms like Na^+^-Ca^2+^ exchangers (NCXs), SERCA pumps and MCU. PMCAs have been found to have an important role in regulating presynaptic calcium level in DRG synapses in the dorsal horn [118]. On cultured DRG, upon small Ca^2+^ loads, PMCAs are responsible for lowering the free intracellular calcium concentration, while calmodulin and PKC signalling pathways take part in the modulation of PMCA-mediated Ca^2+^ efflux [14]. In addition, prolonged depolarisation, sustained action potentials trains and TRPV receptors activation, all leading to increased intracellular calcium concentration, have a priming effect on PMCAs accelerating PMCAs-mediated Ca^2+^ efflux in DRG sensory neurons [119]. Thanks to this central role in intracellular resting calcium concentration modulation, PMCAs may be involved in neuropathic pain generation in primary sensory neurons. This is supported by the fact that upon spinal nerve ligation, an elevate activity of dorsal root ganglia PMCAs results in a decrease of resting cytoplasmic Ca^2+^ levels, contributing to neuronal hyperexcytability [120]

### 4.6. NCXs

Another mechanism to reset the Ca^2+^ concentration to the basal level is represented by Na^+^-Ca^2+^ exchangers (NCXs), membrane proteins present along the central nervous system and sensory neurons, both on their free terminals and cell bodies [121,122]. They can exchange Na^+^ and Ca^2+^ in either direction, depending on their electrochemical gradient and while under basal conditions they extrude calcium and import sodium, when the intracellular Na^+^ concentration is high or with depolarised membrane there is a reversion of the exchange directions with calcium entering and sodium being exported [14]. The reverse mode of NCXs has been associated with neuropathic pain in sensory neurons, following peripheral nerve injury, whereas this reverse mode makes them a possible interesting player in sustained nerve depolarisation associated with nerve damage and more in general in increased neuronal excitability in pathological pain sensitisation conditions [79,123]. Finally, NCXs are located also in the inner nuclear envelope where they may be involved in ER-nuclear calcium signalling and therefore in the regulation of calcium signalling machinery itself [70,124].

### 4.7. Mitochondrial Contribution in Pain Signalling

Mitochondria, beyond their important metabolic role, are one of the sources of calcium signalling also in neurons, where they may almost be considered as a separate system given the intricate networks they can form. They can represent a source of calcium but also a huge buffering system that, if altered or dysfunctional can lead to a modification of the physiological pain signalling system. While in a global view, through MCU and NCX proteins, mitochondria can have a role in the control of neuronal calcium firing amplitude and duration [125,126], they have been identified as regulators of presynaptic calcium transients, controlling neurotransmitter release from sensory nerve fibres in the spinal cord and playing a role in long term potentiation [118,127,128,129]. Indeed, mitochondria were found to be functionally coupled with TRPVs mediated calcium influx and to increase long-lasting elevations in cytosolic calcium concentration [127,129] with a consequent increase of neuronal firing rate and neurotransmitter release [129]. Besides having a role in calcium entry mitochondria are also involved in calcium clearance together with PMCAs especially in sensory neurons and in spinal cord synapses [118].

## 5. Cancer Bone Pain

The bone is a highly complex tissue. It is populated by a broad variety of cell types coexisting in the same environment and strictly influencing each other, in some cases thanks to highly complex signalling niches, as happening for hematopoietic stem cells.

The bone is widely innervated by A-delta and peptidergic C fibres with the great majority of them (>80%) expressing TrkA [130,131] and receives little if any innervation by A-beta fibres and unmyelinated peptide-poor C fibres. The density, pattern and morphology of these two main fibre populations may vary from joint and bone but also between periosteum, cortical bone and bone marrow.

The periosteum, a thin fibrous sheet covering the entire bone, is innervated by A-delta and peptidergic C fibres, organized in a fishbone-like pattern, responsible for the detection of mechanical injury or distortion of the underlying cortical bone [132]. The activation of this sensory network results in the initial sharp, stabbing and arresting pain felt upon kick or fracture, being transmitted by the thinly myelinated A-delta fibres, following the activation of mechanotransducers, ion channels able to detect stretching and pressure stimuli [133]. In physiological conditions, the pain following bone fracture upon movement before an effective stabilisation may therefore be imputable to the normally silent mechanosensitive nerve fibres densely innervating the periosteum. After the fracture stabilisation the initial sharp pain is normally slowly substituted by a dull hutching pain which is most likely originating from activation and sensitization of both the A-delta and non-myelinated C fibres innervating both periosteum and bone marrow. This sensitisation process is driven not only by the activation *per se* of the neuronal circuit but in great part also from the release of algogenic factors by the different cells invading the bone fracture site. This also happens in case of CABP.

The expanding tumour inside the bone activates nociception mechanisms in many different and still unexplored ways [134]. CABP aetiology is multifactorial, being the result of nociceptive nerve compression from growing tumour cells and micro-fractures, together with a complex inflammatory signalling and altered secretome niche, leading to increased bone innervation and to peripheral and central sensitisation [135,136].

It has been proposed that the mass growth in the bone marrow cavity can exert a direct effect upon the periosteum which, being stretched by the increased intraosseous pressure, can activate the mechanosensors of the nociceptive sensory neurons innervating the periosteal surfaces [137]. In the same context, tumour cells invading the internal bone cavity can act directly on the very distal processes of nociceptive fibres compressing and damaging them resulting in neuropathic pain [134,136]. This theory is supported also from clinical evidence where neuropathic bone pain has been attenuated by the administration of gabapentin which, binding to α2δ1 subunit of VOCCS, disrupts their trafficking to the presynaptic sites [98,99] being approved for treatment of neuropathic pain [138] and effective in reducing pain in CABP [139].

Studies in both mice experimental models and cancer patients led to the conviction that hyperalgesia, allodynia and spontaneous ectopic discharges that are perceived as highly painful in humans can originate from this pathological inappropriate sprouting and ectopic remodelling of sensory nerve fibres in the bone [140,141,142,143]. This effect can be explained by the expression of NGF by breast cancer cells and tumour-associated inflammatory, immune and stromal cells like macrophages, mast cells, endothelial cells, lymphocytes, eosinophils and fibroblasts. Since these cells comprise 20% to 80% of cells in breast cancer, altogether they are thought to secrete a significant amount of NGF with a significant role on pathological nerve sprouting [136]. NGF acts directly on the TrkA${}^{+}$ nerve fibres, playing an important role in axonal sprouting. To support this evidence, anti-NGF therapy on a metastatic breast cancer mouse model resulted in decreased neuroma formation and nerve fibres reorganization, preventing allodynia and hyperalgesia [3,144].

Together with NGF, also BDNF has been shown to play a vital role in several cancer types including breast cancer and to be highly upregulated, together with its receptors TrkB and p75 [145,146]. Interestingly, this pathway was shown to be upregulated also in sensory neurons of a CABP animal model, where DRG neurons had significantly higher expression of BDNF and TrkB with direct consequences on the increased pain perception [147] while in the same model administration of anti-BDNF therapy had anti-nociceptive effects.

### Calcium Signalling in Cancer Bone Pain

**Figure 3:**
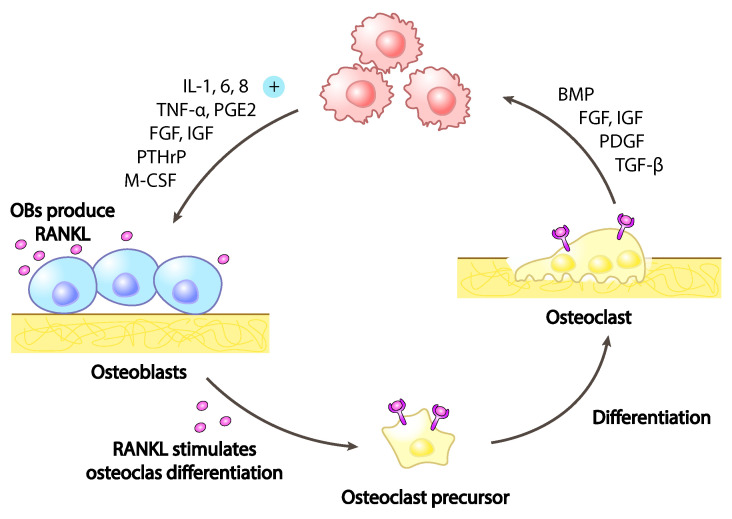
Vicious cycle of osteolytic metastases. Tumoural cells secrete soluble factors as interleukines (IL), tumour-necrosis factor alpha (TNF-α) and parathyroid-hormone related peptide (PTHrP), promoting osteoblasts mediated receptor activator of nuclear factor kappa-B ligand (RANKL) secretion. RANKL stimulates pre-osteoclasts differentiation into fully mature osteoclasts increasing therefore the bone matrix degradation and the release of the soluble factors trapped in it, like transforming-growth factor beta (TGF-β), bone morphogenic protein (BMP), fibroblasts growth factor (FGF), insuline-like growth factor (IGF) and platelet-derived growth factor (PDGF). Finally, these growth factors stimulate tumoural growth resulting therefore in a feed forward vicious cycle.

The presence of breast cancer cells in the bone microenvironment has a direct role on the more intimate bone nature. Breast cancer cells alter the equilibrium between osteoblasts, the bone-forming cells and osteoclasts, the bone-resorbing cells. The secretion from breast cancer cells of signalling molecules like vascular endothelial growth factor (VEGF), interleukin-6 (IL-6), IL-8,IL-11, macrophage colony-stimulating factor (MCSF), receptor activator of nuclear factor kappa-B ligand (RANKL), sonic hedgehog (SHH) and parathyroid hormone-related protein (PTHrP) (Figure 3), results in an inbalance toward osteoclasts hyper-activation with consequent increased bone degradation leading to pathological bone tissue loss and micro-fractures as cause of CABP.

Together with this, the very same bone matrix being degraded, releases growth factors together with Ca^2+^ and H${}^{+}$ ions. While matrix derived growth factors stimulate tumoral cells proliferation, the released protons contribute to the increase of the extracellular environment acidification [2], directly stimulating acid-sensing neuronal receptors and with this the bone tissue innervation [134].

The critical role of osteoclasts resorbing activity in CABP pathophysiology has been clinically confirmed since the administration of anti-resorptive agents as denosumab and bisphosphonates exerts a positive effect on patients affected by solid cancers and multiple myeloma reducing bone pain [148,149]. Together with this clinical evidence, in a study using an experimental animal model was reported the positive effect of direct administration of the RANKL decoy receptor osteoprotegerin (OPG) on bone pain suppression. Osteoclasts maturation inhibition with OPG, undermines the excessive tumour-induced bone destruction, limiting neuronal nociceptors activation and damage. Finally, the reduced bone resorption prevent neurochemical changes in the spinal cord involved in the generation bone cancer pain and in its chronicization [150].

**Figure 4:**
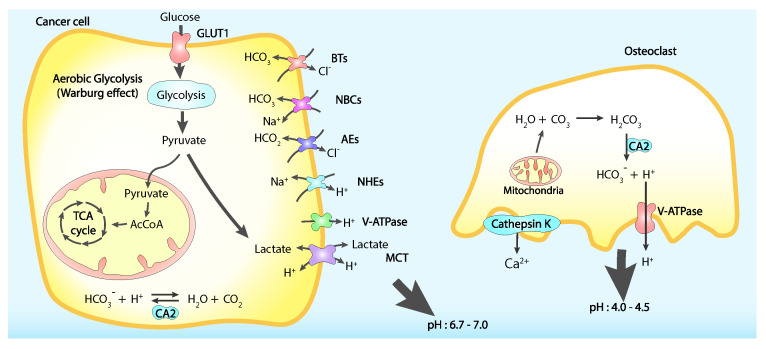
Mechanisms of extracellular acidification: Tumoural cells secrete H^+^ ions and import HCO_3_^−^ to counter the intracellular acidification resulting from the increased glycolysis (Warburg Effect). This actively regulated process involves many plasma membrane proteins like HCO_3_^−^ transporters, co-transporters and exchangers, proton pupms and exchangers, and monocarboxylate transporters (MCT). At the same time osteoclasts actively secrete protons to degrade the minaralized bone matrix acidifying the bone extracellular environment. Bicarbonate transporters (BTs), sodium-bicarbonate co-transporters (NBCs). anions exchangers (AEs), sodium-hydrogen exchangers (NHEs), vacuolar proton-translocating ATPase (V-ATPase), carbonic anhydrase 2 (CA2), glucose transporter 1 (GLUT1). Adapted from [134].

Another source of extracellular environment acidification is represented by the tumoral cells themselves. The elevate aerobic glycolysis on which these cells rely to circumvent mitochondrial oxidative phosphorylation [151], metabolic state known as Warburg effect, produces substantial amounts of lactate thus reducing intracellular pH through the consumption of glucose. To avoid an excessive cytoplasmic acidification tumoral cells actively extrude lactate and protons, therefore contributing to the lower pH of the surrounding medium (Figure 4) [152,153,154].

Nociceptive neurons are sensitive to pH variations, where two identified sets of dorsal root ganglia (DRG), have been shown to have different ways to reply to different acidic stimuli and many mediators of these behaviours have been identified like TRPV1, acid-sensing ion channel 3 (ASIC3), TRPV4, TRPA1 and TRPM8 [134]. TRPV1 role in CABP has been extensively characterised. When activated by pH below 6.0, TRPV1 generates sustained cation currents but in mild acidosis conditions with pH between 6 and 7, and in presence of inflammatory mediators like prostaglandins and NGF it becomes more sensitive to other noxious stimuli [155]. In addition to this, transforming growth factor beta 1 TGF-β1 and insulin-like growth factor 1 IGF-1, stored in the bone matrix and released upon cancer-induced bone resorption by osteoclasts, are responsible for TRPV1 expression and activity upregulation [156,157]. Consequently, to these findings, TRPV1 inactivation and gene disruption result in reduced CABP [158]

Consistent with this discovery, the environmental acidification is considered as another main player of the CABP actively participating in noxious signalling augmentation. Targeting these mechanisms could therefore not only alleviate CABP but also arrest cancer cell growth and kill cancer cells elevating the breast cancer cell intracellular pH.

The increase of the nociceptive stimuli and the altered bone microenvironment at the axonal peripheral site, drives consistent changes also at the neuronal cell body and nervous ganglia located in the spinal cord. While the mechanisms underlying these changes are still widely unexplored due to complexity of the CNS, some evidences have been reported. In cancer-induced bone pain DRG and dorsal horn neurons show increased expression of c-FOS and dynorphin, together with markedly hypertrophic spinal astrocytes and upregulation of galanin and activating transcription factor 3 (ATF3), considered as markers of DRG nerve damage, but no increase on substance P and CGRP has been reported, differently from inflammatory and neuropathic pain [159,160]. In the spinal cord of cancer-induced bone pain models, the neurons show increased probability of response to low-threshold peripheral stimuli and enhanced firing to evoked excitations, reflecting a general hyperexcitability of the neurons. This seems to result from the increased wide dynamic range neurons in the superficial dorsal horn over the nociceptive specific ones, with a consequent broadened receptive field. In turn, this results in the chronic sensitisation of the central nervous system, leading to the enhanced frequency of painful events and dull pain persistence [161,162,163].

## 6. Conclusions

Calcium signalling exerts a pivotal role in many cellular mechanisms related to breast cancer disease like cancer progression, metastatisation, drug resistance and pain, especially after breast cancer cells homing to the bone. While the calcium signalling tools are shared, their role can deeply vary depending on the different cell types and contexts. Understanding the role of calcium in these mechanisms is extremely complex. On the other hand, cancer-associated bone pain is a pathology with a high complexity level for the plurality of signalling pathways and cell types involved. This subject becomes even more complex when it comes to uncovering the mechanisms of central nervous system sensitisation and pain chronicization. Despite many researches only focusing on each particular aspect, cancer associated bone pain is a multi-factorial disease requiring studies with a broader point of view, trying to consider all the cellular players together. A deeper understanding of the calcium mediated connections amongst the plurality of the cellular players involved may be relevant to unveil the full disease complexity and finding new therapeutical targets both for cancer progression and pain control.
